# Non-labeled lensless micro-endoscopic approach for cellular imaging through highly scattering media

**DOI:** 10.1042/BSR20170027

**Published:** 2018-01-25

**Authors:** Omer Wagner, Aditya Pandya, Yoav Chemla, Hadar Pinhas, Irina Schelkanova, Asaf Shahmoon, Yossi Mandel, Alexandre Douplik, Zeev Zalevsky

**Affiliations:** 1 Faculty of Engineering and the Nanotechnology Center, Bar Ilan University, Ramat-Gan 5290002, Israel; 2 Institute for Nanotechnology and Advanced Materials (BINA), Bar Ilan University, Ramat Gan, Israel; 3 Physics Department, Ryerson University, Toronto, Canada; 4 Faculty of Life Sciences, School of Optometry and Visual Science, Bar Ilan University, Ramat Gan 5290002, Israel

**Keywords:** epithelial cells, Fiber optics imaging, imaging techniques, Imaging through Scattering media, retina, Superresolution

## Abstract

We describe an imaging approach based on an optical setup made up of a miniature, lensless, minimally invasive endoscope scanning a sample and matching post processing techniques that enable enhanced imaging capabilities. The two main scopes of this article are that this approach enables imaging beyond highly scattering medium and increases the resolution and signal to noise levels reaching single cell imaging. Our approach has more advantages over ordinary endoscope setups and other imaging techniques. It is not mechanically limited by a lens, the stable but flexible fiber can acquire images over long time periods (unlike current imaging methods such as OCT etc.), and the imaging can be obtained at a certain working distance above the surface, without interference to the imaged object. Fast overlapping scans enlarge the region of interest, enhance signal to noise levels and can also accommodate post-processing, super-resolution algorithms. Here we present that due to the setup properties, the overlapping scans also lead to dramatic enhancement of non-scattered signal to scattered noise. This enables imaging through highly scattering medium. We discuss results obtained from *in vitro* investigation of weak signals of ARPE cells, rat retina, and scattered signals from polydimethylsiloxane (PDMS) microchannels filled with hemoglobin and covered by intralipids consequently mimicking blood capillaries and the epidermis of human skin. The development of minimally invasive procedures and methodologies for imaging through scattering medium such as tissues can vastly enhance biomedical diagnostic capabilities for imaging internal organs. We thereby propose that our method may be used for such tasks *in vivo*.

## Introduction

Current non-invasive imaging approaches with high spatial resolution such as single/multiple photon fluorescence or confocal fluorescence micro-endoscopy can be used *in vivo* [1–3]. However, they are only applicable at shallow interrogation depths, thus prohibiting deeper examination. Approaches using MRI, computed tomography (CT), high and low frequency ultrasound (US) allow deeper penetration but they are limited when long periods of time and/or high spatial resolution imaging are needed. To respond to this need, alternative technologies consisting of minimally invasive micro-endoscopes using optical fibers have been developed to achieve high spatial resolution while permitting deep insertion of the device into the target area, and long-term monitoring of the implant.

Typically, the commercial fiber consists of numerous cores bundles, each of which acts like a fiber in itself. Bundle configurations known as multicore fibers (MCF) eliminate core-to-core light coupling mainly through distance. When each core is in a sufficient length from its neighboring cores, a pixelated image is generated. The outcome, however is that the image resolution is damaged and pixelation artifacts are generated. Many micro-endoscope configurations have introduced graded index (GRIN) microlenses [1,4] that facilitate the collimated collection in a bid to improve resolution and enable a location farther away from the sample. However, using the GRIN lens limits the mechanical rigidity and length of the endoscope as does miniaturizing its diameter to enable deeper penetration and lessen tissue damage.

To overcome these obstacles, many approaches have turned to multimode fibers (MMF) [5–7]. However, MMF tend to scramble the information transmitted through them both in space and time [8]. To rectify this problem, complex approaches such as optimization algorithms; digital phase conjugation or transmission matrix are required to shape the wave front [7]. However, bending the fiber changes the relative mode propagation, forcing a new calibration. One alternative to MMF are single-mode fiber bundles (SMFB). SMFB methods reach diffraction limit by using resolution enhancement approaches employing scanning heads with lenses, a spectral disperser, or speckle correlations [9,10] which are less sensitive to the above limitations.

In both MMF and SMFB, lensless bundles enabled the development of methods that project light through the bundle to illuminate the sample. The reflected light is collected through the same bundle and is used to image the sample [8,11].

These have also led to significant progress in the construction of fiber optic confocal micro-endoscopes which can perform optical sectioning. Some methods require beam-scanning systems at either the proximal or distal end of the fiber bundle [12,13], whereas new MMF structured light scanning methods are indifferent to bending [14–16]. Although such systems can perform optical sectioning, they need complex active modulated illumination and staining of the sample with a fluorescent contrast agent. Current technologies have a penetration depth of less than 150 µm that cannot deal with scattering medium between the fiber and the sample [14–16]. Another limitation of these light scanning methods is their speed, which can take between roughly 5 Hz for a 36×36 pixel image up to several minutes [16]. A recent speckle correlation technique was able to conduct optical sectioning without staining, but the imaging was only achieved through sparse targets [17].

Recently, Shahmoon et al. [18] described a lensless SMFB that can overcome many of the above-mentioned limitations *in vivo*. The 200-µm fiber consists of ~5000 inner cores and does not need the large distal optics that are usually required for selecting an imaging plane away from the tissue damaging tip [16]. It was reported to have a working distance of up to 0.5 mm.

Here, we use the same SMFB and exploit the working distance to conduct a scan with small translations between acquisitions followed by post processing steps. This adds new capabilities to our previous work [18] as the resolution and signal to noise levels are raised.

Importantly, the densely located cores constitute an advantage for imaging through highly light scattering media such as biological tissues. Specifically the core constrains a narrow sampling region while the large working distance causes scattered noise to have large shifts in comparison with the small displacements of the fiber. This promotes the collection of non- scattered or low-scattered light and scarces stray light.

After achieving an adequate signal to noise ratio, the target can then be recovered with enhanced resolution using geometrical super resolution methods (G-SR) [19–21] based on the scanning methodology. These methods are highly efficient given the fiber’s small core size and pitch, but still have a low fill factor as compared with other reported fibers.

We describe the ways in which this technique in conjunction with our modified SMFB increases the signal to noise levels such that a single cellular ARPE cell can be imaged without labeling or phase microscopy. Further applications are illustrated in imaging slices of rat retina. To show how cellular imaging can be achieved through deep scattering layers, we describe how we successfully imaged a 50-µm diameter microchannel filled with hemoglobin covered by an intralipid layer that matched the scattering properties (µ_s’_) of the epithelium [22–24], at thicknesses ranging up to 360 µm in a polydimethylsiloxane (PDMS) matrix. The image was collected by scanning the area of interest in both the transmission and reflected modes.

## Methods

### Experimental system

**Figure 1:**
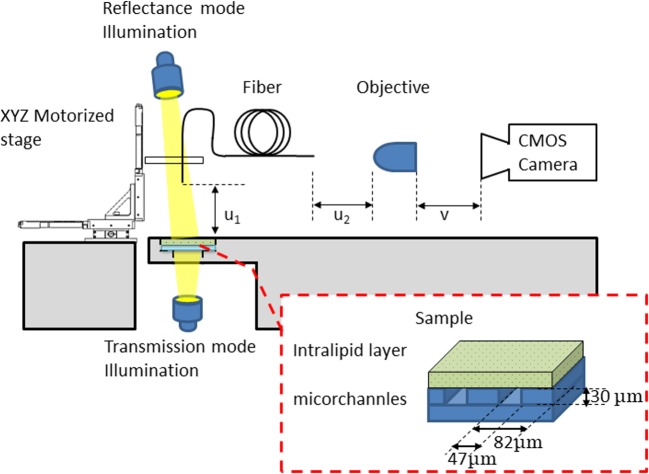
Multicore micro-endoscope imaging system Incoherent white light LED or a partially coherent 532 nm laser passing through a rotating diffuser illuminates the sample from underneath (transmission experiment) or the top (reflectance experiment). The light was transmitted or reflected from the sample covered by intralipid layers. The light enters the scanning end of the multicore fiber that can be moved using a 3-axis motorized stage to scan the sample. The external end is fixed and the light is collected using an imaging lens and a CMOS sensor. Magnification and distance of the fiber end from the sample are determined by the relation between the distances u_1_, u_2_, and v. u_1_ is the distance between the distal end of the multicore fiber and the sample. u_2_ is the distance between the external end of the fiber and the objective. v is the distance between the objective and the CMOS camera.

Our experimental setup is shown schematically in Figure 1. An incoherent white light emitting diode (LED) or a partially coherent 532 nm laser passing through a rotating diffuser illuminates the sample from two positions: below the sample for the transmission mode and above it for the reflectance mode. The scanning end of the optical micro-endoscope is located above the sample. A V-groove holder, axially positioned, holds this end and translates using 3-axis motorized stage. The external end of the optical micro-endoscope is positioned and aligned within the focal plane of an objective (Motic, E-FN PLAN 20X), which in turn is coupled with a CMOS camera (Basler acA2000-50).

### Samples

For calibration, a USAF power test target 1951 was tested for calibration and assessment. The target was imaged with and without diffusive medium layers.

Three types of biological samples were tested. To assess whether our method can be used for cellular imaging, ARPE-19 cells (ATCC® CRL-2302™) were grown in a fibroblast medium and were seeded on coverslips in 24-well plates. The medium contained 90% DMEM (Biological Industries), 10% FBS (Biological Industries), 1 mM glutamine (Biological Industries), 50 μg/ml streptomycin (Biological Industries), and 50 units/ml penicillin (Biological Industries). The medium was changed once every 2 days. Two days after seeding, the living cells were visualized with an inverted microscope using the phase-contrast modality (Olympus -CKX41). The cells were then rinsed with PBS (0.14  M NaCl, 2.5  mM KCl, 0.2  M Na_2_HPO_4_, 0.2  M KH_2_PO_4_) and fixed with 4% paraformaldehyde for 25 min at room temperature, and then rinsed with PBS. After fixation, the cells were ready for visualization.

To test the imaging of a complex biological system, 5-µm thick sections of rat retina were imaged *ex vivo* on fixated and paraffin-embedded retinal sections, as done in [25].

To test imaging beyond a diffusive medium, we used a microfluidic phantom based on PDMS substrate. To create the phantom, microchannels were carved into the material, creating 47-µm wide grooves. The groove height was 30 µm and each groove was spaced 82 µm from its neighboring grooves. We filled the microchannels with hemoglobin extracted from red blood cells (160 g/l, Sigma–Aldrich, U.S.A.), and covered it with layers of diffusive medium.

To create the diffusive medium layers, a solution of intralipid (I141, Sigma–Aldrich Inc, U.S.A.) was diluted in deionized water to obtain 1 and 2%. This concentration was selected to have a scattering coefficient μ_s’_ of 1.3 [mm^−1^] at λ =545 nm that matches the scattering properties of the epithelium [22–24]. The layers tested had thicknesses of 240, 320, and 360 µm.

### Imaging SMFB

The SMFB consists of 5000 inner cores, fabricated from polysterene (PS) with a refractive index of 1.597 (λ =545 nm). The clad was composed of poly methyl methacrylate (PMMA) with a refractive index of 1.495 (λ =545 nm), so it has an NA of ~0.56 at an illumination wavelength of 532 nm. The core’s size was 835 nm in diameter with a 2.3 μm pitch between nearest cores. Although the fiber has a diameter of 200 µm, we used an active field of view (FOV) of ~100 μm × 100 μm.

### Experimental procedure

We defined the relative distance between the distal (at the sample side) end of the multicore fiber and the sample as u_1_. The distance between the external end of the fiber and the objective was defined as u_2_. The distance between the objective and the CMOS camera was defined as v. These distances were empirically predetermined so that u_1_, which was the physical working distance, allowed movement above the intralipid layers. As a first estimation, we assumed that u_1_ =0.1 mm, to reach the magnification M =25 we calculated from eqn 1 that u_2_ =1.532 mm, v =40.8 mm.

(1) $$M\;=\;\frac{f_{objective}}{f_{objective}-\;(u_{1}\;-\;u_{2})}\;=\;\frac{v\;-\;f_{objective}}{f_{objective}}$$

We placed intralipid gel layers of different widths on top of the microchannel sample and mounted it on the stage. The scanning end of the optical micro-endoscope was lowered. By setting v =40.8 mm, we focussed on the sample to reach a theoretical 0.1 mm distance above the sample surface (scattering layer or the top of the grooves). Next, we applied small corrections to the u_1_ and v values to reach a proper scan.

Prior to each experimental procedure, some processed images were utilized as reference images. These background images without a sample (for all samples – ARPE cells, rat retina, microfluidic and USAF target, with and without the scattering layers) were illuminated in the transmission and reflection modes while applying different exposure times related to the exposure times that were used in the later experiment (30–250 ms). The sample was then scanned at 10–50 μm steps, taking several images with different exposure times in each step for image processing techniques such as flat-field correction [26].

Because the scanned images were partially overlapping, interpolation between them enlarged the region of interest, and reduced temporal noise. Furthermore, each translation introduced new data in the overlapping area that could then be extracted using G-SR algorithms.

This procedure had another advantage, due to the 0.1-mm working distance and small imperfections in the distal end movement relative to the sample’s surface. Non-scattered signal is shifted according to the small translations while scattered noise shifted away from this position. This was the main reason that enabled imaging through turbid medium.

### Data analysis

The first step of our image processing was to determine the allocation of the cores from the image processing reference images, as described at the end of ‘ Samples’ section. A corrected version [27] of the classical particle tracking algorithm developed by Crocker and Grier [28] was applied to these images to compute the rough co-ordinates for the core positions as a first approximation. Then we calculated a more accurate estimation for the center position by fitting the intensity of each core to a Gaussian profile. The core location was used to screen the fiber sampling pixels with valid data against the pixels that imaged the clad or image fault cores.

The second image-processing step addressed differences in the cores response to illumination. A variable exposure flat-field correction methodology [26] was applied. This type of algorithm does a better job of eliminating fixed-point noise, particularly with high spatial frequencies or incident exposures, than non-variable ones. The various exposure reference images were used to calculate the variables needed for this method.

After the extraction, the core location and flat-field correction parameters, the following image-processing steps were applied on the images of a sample scan:

A flat-field correction was applied to each image. The displacement size and direction between two consecutive scan images was calculated using a correlation method based on the one described in [29], taking the motor step size as the first guess. Using the displacement values, the images were stitched to produce a ‘whole image’ with an enlarged FOV.

In the following step G-SR procedure ([19–21] mentioned in ‘ Introduction’ section) was applied, based on the fact that the images were acquired in a partially overlapping pattern as outlined below. In step (1) the ‘ whole image ’ represents a processed imaged stitched from the entire set of single images of the scan. The core location of each single image was marked in the whole image. In step (2) the data from all core locations were extracted from the whole image, and an image where each pixel represented a core was made. In step (3) the resulting image was Fourier transformed and divided in the frequency plane by the frequency response image of a single core in that plane. The result was inverse transformed to obtain the final image.

G-SR algorithm:


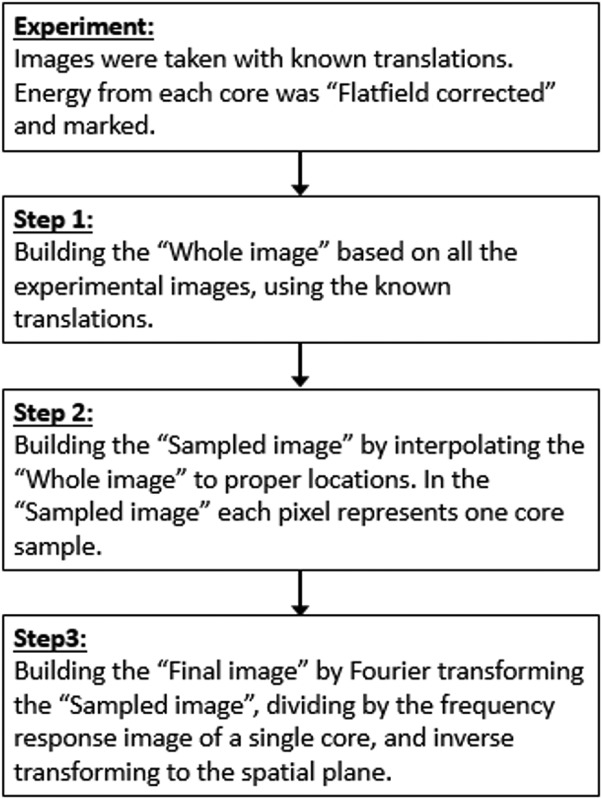


The theoretical resolution limit of the system without applying G-SR was twice the pitch between the nearest cores (4.6 μm). After applying stitching and G-SR, the resolution was limited by the size of the core and the accuracy of the relative motion estimation. Analysis of the final image was quantitated using measurements of the line width full width half maximum (FWHM), and its contrast defined as:

(2) $$C\;=\;\frac{Max(S)\;-\;B}{Max(S)\;+\;B}$$

Where S is the line value and B is the background level.

## Results and discussion

**Figure 2:**
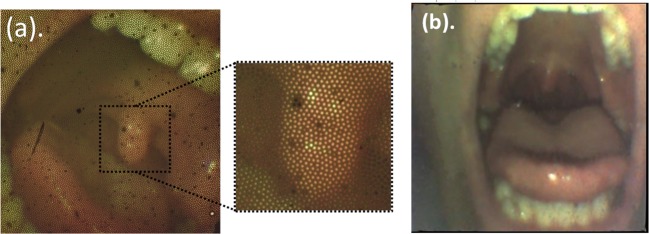
Image of a mouth experimentally obtained from the micro-endoscope (**a**) Raw data, and (**b**) after image processing.

Before we present the results of the added SMFB capabilities, pushing to its resolution limits and extracting an image via scattering medium, it is important to note that this fiber can be used as a very good quality micro-endoscope that can provide high quality color images of internal organs. For instance, Figure 2 shows an image of the internal part of a mouth taken with the SMFB without processing (raw data) in Figure 2a and after applying a properly developed image processing tool kit in Figure 2b. The image processing algorithm that we have developed included allocation of the positions of the cores of the fiber and then building an image constructed only from the values of the cores. Right after we have performed a spatial interpolation to generate a smooth image without seeing the artifacts that the cores generate. We have also performed color correction by normalizing the colors to the white level in order to obtain a more balanced and more natural colors in the interpolated image. We also refer the reader to source [18] where *in vivo* imaging of a rat brain microvascular area was done.

**Figure 3:**
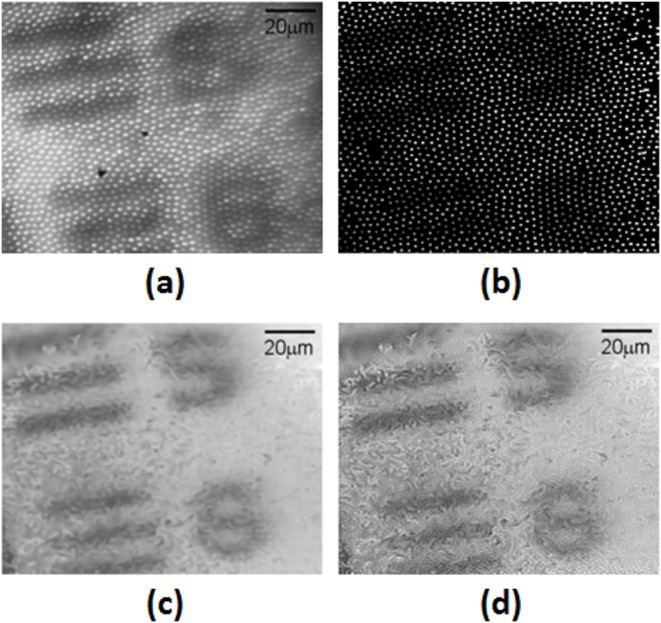
Imaging of USAF power test target 1951 group 5, element 5 (upper part of the image) and 6 (lower part of the image) The Illumination is set in the reflective mode. For each single image of the target (**a**), the core positions are extracted (**b**), and the relative motion between the images is calculated. All the images are then stitched and interpolated (**c**), the G-SR algorithm is applied (**d**). All images are equally scaled.

Demonstration of the image analysis process is shown in Figure 3 which shows the results of the step-by-step analysis process of imaging the USAF target 1951 (group 5), elements 5 (9.84 µm width lines) and 6 (8.77 µm width lines). A raw image is displayed in Figure 3a. Extraction of the cores positions and flat-field correction parameters was done on a previous calibration image. The target was scanned and data from the core position in each image was extracted and corrected, an example is shown in Figure 3b. The 20-μm translation scan was stitched and interpolated from the scan series (Figure 3c); then the G-SR algorithm was applied. The final processed image is shown in Figure 3d.

**Figure 4:**
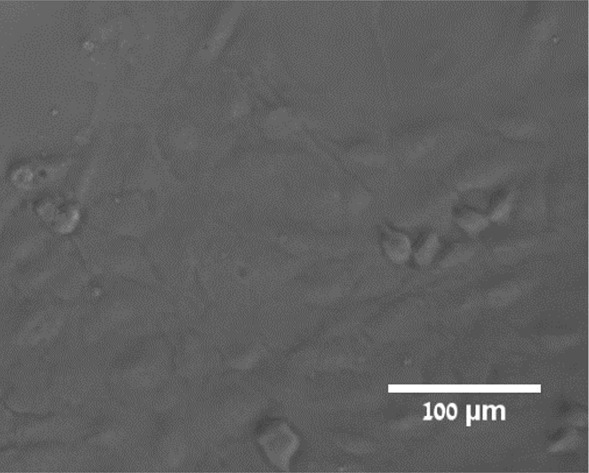
ARPE cells image taken with an inverted microscope using the phase contrast modality (Olympus -CKX41) with ×20 magnification

As shown in Figure 3. the resolution scale reached using the fiber is sufficient for cellular imaging. However, the sharp contrast and absorbance of the USAF target is different from the optical signal of a single cell. The signal due to the cell’s pigmentation light absorption is very faint. To assess our system’s cellular imaging capabilities, we imaged non-labeled ARPE cells. Using conventional microscopy, phase contrast imaging is required to acquire such a signal. Hence, the sparsely deposited ARPE cells samples were imaged prior to the experiment using an inverted microscope with phase contrast modality and a ×20 objective as shown in Figure 4.

**Figure 5:**
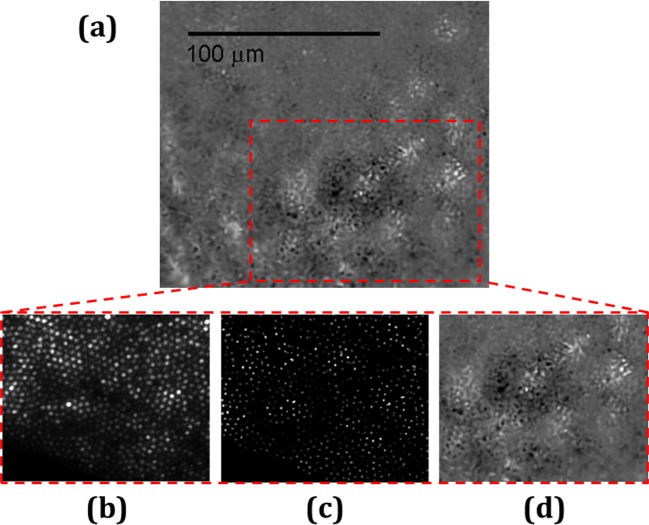
ARPE cells image taken using our SMFB and post-processing The image after scanning and post-processing is shown in (**a**) and a magnification of the red dashed rectangle is shown both to a single raw image (**b**), the same image after cores intensities extraction and flat-correction (**c**), and the magnified area after final image processing where the ARPE cell is now resolved (**d**).

A scan of the same slide shown in Figure 4 (but not the same area) using the fiber is shown in Figure 5a. Taking acquisitions with 20-μm translations allowed image stitching and applying G-SR algorithms. Importantly, the source of illumination which gave the highest contrast was a partially coherent 532 nm laser, generated simply by passing the light through a rotating diffuser in the transmission mode.

For better understanding of the algorithm process, the red dashed rectangle area in Figure 5a is zoomed. The intensities of a raw image in Figure 5b are highly fluctuating and noisy which makes it very difficult to get a meaningful signal from the image. Regions of similar levels and border features are exposed after the cores intensities extraction and flat-correction as shown in Figure 5c. This image also helps to understand how narrow the fiber sampling is, and to estimate the sampling density in comparison with the cells. The ARPE cells can clearly be distinguished from the background by applying the image stitching and G-SR algorithms as shown on Figure 5d. Their morphology could further be examined with higher resolution.

To assess the fiber’s capabilities to image a complex biological system with micron-sized and cellular features we examined a non-labeled 5-µm thick sliced rat retina section. We found again, that partially coherent light illumination in the transmission mode gave the highest contrast results. We examined samples with and without a coverslip.

We compared the morphology between images of the section that were taken with an inverted microscope using the phase contrast modality (Olympus -CKX41) against the images processed from the fiber acquisitions.

**Figure 6:**
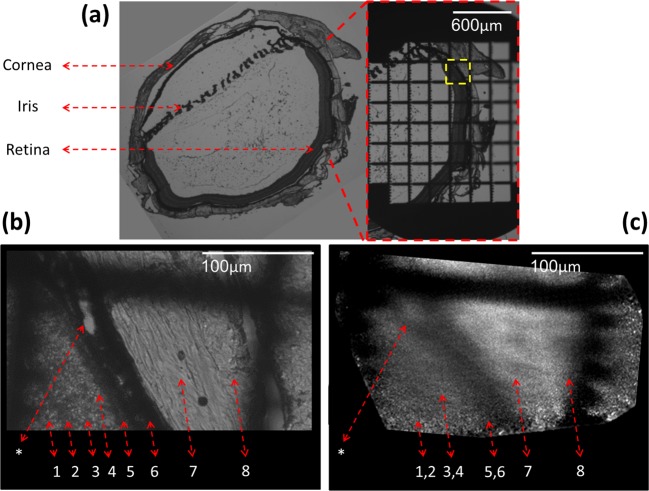
Full algorithm processed imaging of rat retina (**a**) Rat retina 5-µm thick sliced section images taken using the phase contrast modality (Olympus -CKX41) with ×4 magnification, unmarked slice on the left and the same slice marked by a grid target on the right, (**b**) ×20 phase contrast imaging of the dashed yellow rectangle. (**c**) Fiber scan image of the same area. The asterisk and numbers 1–9 label: choroidal blood vessel (*), inner nuclear layer (1), outer plexiform layer (2), outer nuclear layer (3), outer segments (4), RPE – pigmented epithelium (5), choroid (6), sclera (7), and conjunctiva (8).

To validate that the imaging is done on the same location, the retina slices were imaged with a ×4 magnification, than marked using a binary grid target (Maxtaform reference finder grids, Ted Pella, Redding, CA), and imaged again. The process is shown on Figure 6a where the whole unmarked rat eye slice is shown on the left side and the marked region is shown on the right side. Both images are scaled the same.

A location marked by a yellow dashed rectangle on the right side of Figure 6a was chosen, scanned using the fiber and imaged with phase contrast ×20 magnification.

The phase contrast image is shown on Figure 6b where the different retinal layers can be identified and numbered by the labels under the dashed red arrows. A distinct choroidal blood vessel (marked by an asterisk) is clearly seen. The inner and outer nuclear layers (2 and 4, respectively), show similar gray level and are divided by the darker outer plexiform layer (3). The photoreceptors’ outer segments can hardly be seen as a narrow strip (5), slightly brighter than the outer nuclear layer. The retinal pigmented epithelium (6) is darker than the retinal layers and is followed by the choroid which is the darkest layer, because the existence of pigmented cells. The sclera and conjunctiva (8 and 9, respectively) are bright layers showing slight different morphology, as a result of different arrangement of collagen layers in these layers.

The fiber scan image, scanned with 10-μm translations at the same square area found using the grid target, is shown in Figure 6c. Note that the square’s inner hole width is 210 µm.

The scan succeeded to resolve the 10-μm width choroidal blood vessel (marked by an asterisk). The inner nuclear layer and the outer plexiform could not be resolved from each other but were significantly darker than the outer nuclear layer. Similarly, the retinal pigmented epithelium and the choroid were dark and could not be resolved from each other. In contrast, the sclera could be resolved from the darker conjunctiva.

**Figure 7:**
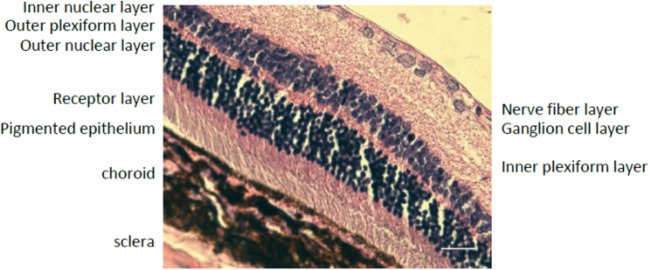
Hematoxylin and Eosin dyed rat Retina imaged with a fluorescent microscope.

To verify the results, a fixated and paraffin-embedded retinal section was Hematoxylin and Eosin dyed as in [30]. The imaging results with a ×20 fluorescence microscope are shown in Figure 7 that depicts the inner layers of the 5-µm thick sliced rat retina section.

We move from quality results that present the setup capabilities in imaging biological systems down to the cellular size, to results that present the article main scope, which is the ability to image beyond scattering media.

To quantitatively examine the setup, two types of line targets were used, USAF targets and PDMS microchannels filled with hemoglobin. The line targets were covered by intralipids with reduced scattering coefficient μ_s’_ of 1.3. Channels’ width and signal contrast were measured as defined in ‘Data analysis’ section as a figure of merit.

Imaging beyond the scattering layer was done using incoherent white LED following the procedure detailed in the ‘Experimental procedure’ section, the calibration that allowed a good working distance from the object without contacting the intralipid layers was found empirically. The relative distances between the external fiber ends, the objective and the CMOS camera determined the magnification to be 26.3.

Imaging of a USAF test target was done to get a low noise measure of the relation between image deterioration to diffusive layers thickness. Transmission mode imaging of 49.61 µm width lines target (USAF test target 1951 group 3, element 3) was chosen, matching line widths of the microchannel sample phantom for later comparison.

**Figure 8:**
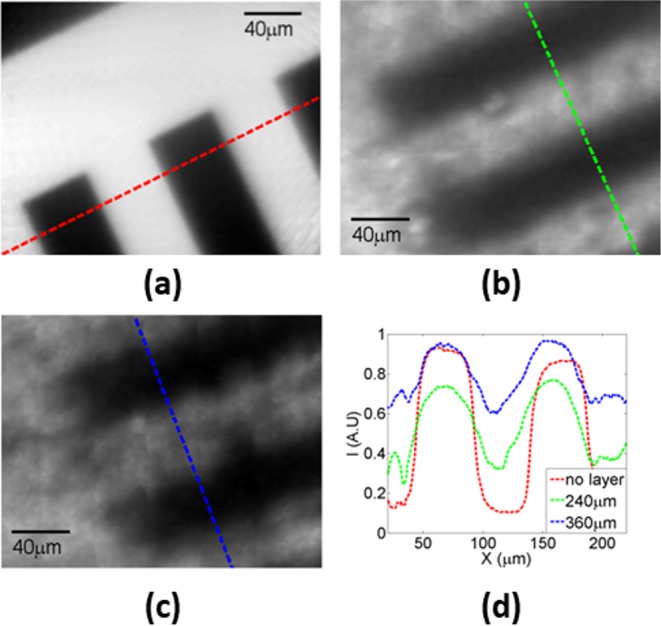
Full algorithm processed imaging of USAF test target 1951 group 3, element 3 The illumination is in the transmission mode. Without scattering layers (**a**), the imaging had very sharp contours, high contrast values, and a resolution of ~1 µm compared with the calculated feature widths extracted from several projected lines (such as shown in dashed red) with the target values. The results of adding 240 µm width (**b**) and 360 µm (**c**) width diffusive medium show the resolution and contrast deterioration. (**d**) Indicates the distribution of intensity I(x) along the dashed lines in (a), (b) and (c), the corresponding colors are matched.

Scanning with 50-µm translations, the USAF target was imaged without, with 240-µm and with 360-µm thick intralipid diffusive layers. The results are shown in Figure 8a–c respectively.

A comparison between the projected intensity distributions along the line profile is shown in Figure 8d. Without any layer, a sharp 0.81 contrast value contour with 49.8 µm line width was measured. Adding the layers the local contrast started to deteriorate, smearing the image. With 240 µm thick layer, the contrast value dropped to 0.6 and the line widths broadened to 52 µm. After increasing the layer thickness to 360 µm, the line widths increased to 57 µm and the contrast value fell to 0.3. Note the broadening of the lines and the increase in the signal to background level values as the diffusive layers are added.

**Figure 9:**
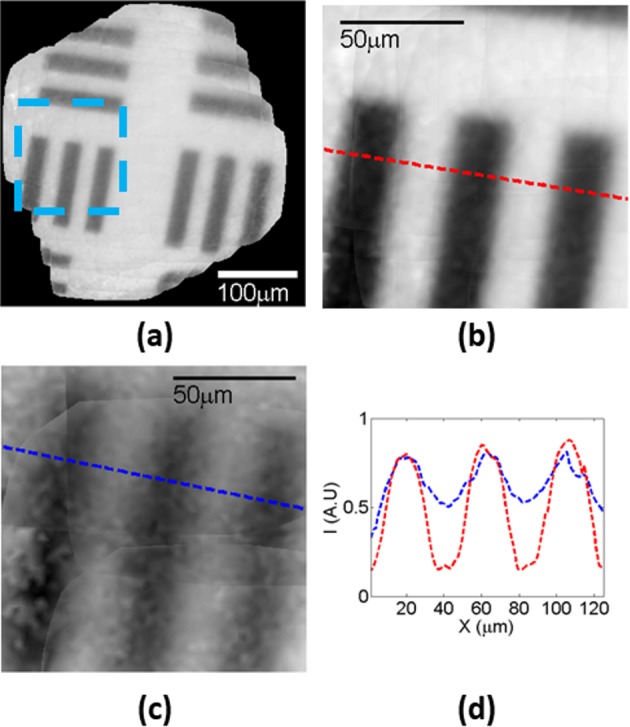
Full algorithm processed imaging of USAF test target 1951, group 4, element 4 The illumination is in the reflection mode. Without any scattering layer (**a**), the imaging had sharp contours and high contrast values and a resolution of roughly 1 µm. Imaging of the light blue area image in (a) is zoomed in and shown in (**b**) and the same area is zoomed and presented in (**c**) showing the results of applying the 240 µm width scattering medium. (**d**) Indicates the distribution of intensity along the dashed lines in (b) and (c), and the colors are matched.

To evaluate the setup imaging beyond scattering layers in a more challenging configuration, results in reflection mode imaging of a target with 22.1 µm width lines (USAF test target 1951 group 4, element 4) are shown in Figure 9. Scanning was done using 30 μm translations.

Imaging without a layer was sharp and the stitching algorithm performance can be evaluated from the large stitched FOV shown in Figure 9a.

Zooming on the area marked by a blue dashed rectangle, the imaging results without a diffusive layer are shown in Figure 9b. The line width measurement was 22.3 µm with a contrast value of 0.72, a sharp contour with a slightly lower value than the result measured in the transmission mode. Adding a 240-µm thick diffusive layer and zooming on the same area, the line widths broadened to 25 µm, and the contrast values deteriorated to 0.3 (Figure 9c). This is similar to the imaging performance done in the transmission mode at the 360 µm wide diffusive layer. The main reason is the fact that in reflection mode the light passed twice through the scattering medium.

A comparison between the projected intensity distributions along a line profile is shown in Figure 9d. It clearly shows that the setup succeeded to image beyond the 240-µm thick diffusive layer. While the lines broadening from 22.1 to 25 µm is rather low, this means that the resolution was not severely damaged. The main concern is in the signal to background level values which dropped.

Imaging the PDMS microchannels filled with hemoglobin was done to get a measure of the relation between image deterioration to diffusive layers thickness in the conditions mimicking consequently blood capillaries and the epidermis of human skin. The sample was scanned with 30-μm translations in transmission mode.

**Figure 10:**
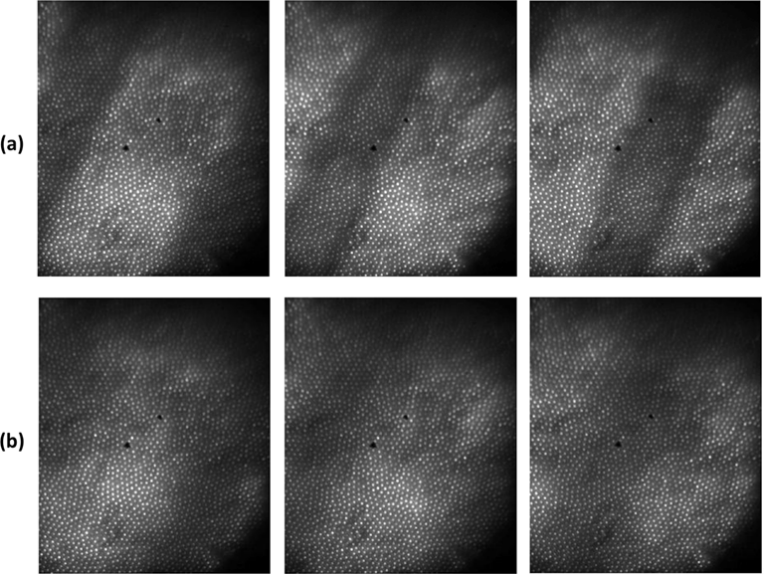
Raw images of hemoglobin-filled capillaries scan with transmission mode illumination Three sequential acquires without any diffusive layer are shown in row (**a**). Three sequential acquires with the capillaries covered by a 240-μm thick intralipid based light scattering medium are shown in row (**b**).

Even without the diffusive layers, there are difficulties to image the weak signal in the noisy environment as can be seen in Figure 10 row (a) where three sequential scan raw acquires without any diffusive layer are shown. However, small motor mismatches in the scan can be easily overcome correlating between the sequenced images and image stitching tremendously increases the signal to noise values.

The effect of adding 240-µm thick diffusive layer on top of the capillary can be seen in Figure 10 row (b) where three sequential scan raw acquires are shown. Buried under the diffusive layer, the signal blurs and a vast amount of scattered noise enters the image severely disrupts feature extraction. This damages the function of motor mismatches correction and stronger algorithms are needed in order to stitch the images in a correct manner that will increase signal to noise ratio while maintaining the resolution.

**Figure 11:**
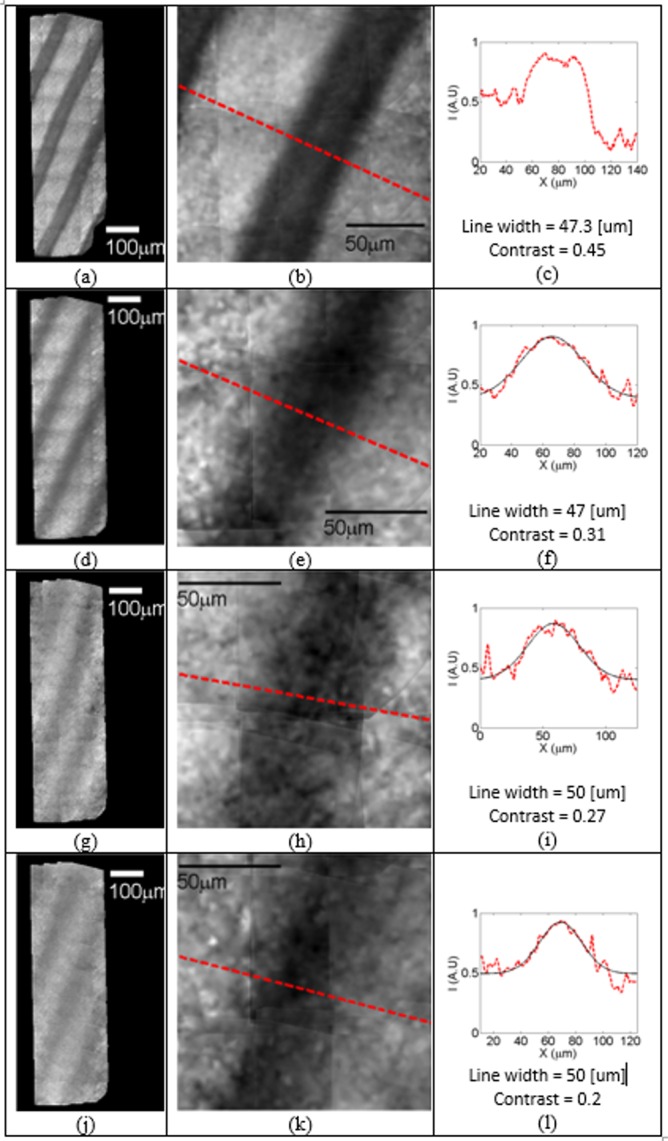
Full algorithm processed imaging of hemoglobin-filled capillaries covered by the intralipid based light scattering medium The illumination is in the transmission mode. The results are shown for scattering layer depths of 0 (**a**–**c**) 240 (**d**–**f**), 320 (**g**–**i**), and 360 (**j**-**l**) µm respectively. For each row, the whole scanned areas (left), zoomed image (central), intensity distribution along the red line (right) are shown.

The results imaging for the hemoglobin-filled capillaries are presented in Figure 11. For each row, the whole scanned area is shown on the left, a zoomed image is shown in the middle and the intensity distribution along the red line in the zoomed image is shown on the right.

Imaging without any layer (a–c), yielded 47.3 µm line width with a contrast value of 0.45. Adding a 240-µm thick diffusive layer, 47 µm line width with a contrast value of 0.31 was measured (d–f). Increasing the thickness to 320 and 360 µm the line broadened in 3 μ and the contrast dropped to 0.27 and 0.2, respectively. In all cases the lines could still be distinctly differentiated from the background.

Consistently, the contrast values are around half the value measured with the USAF target in transmission mode (Figure 8), which means that the same deterioration to layers thickness applies to both targets and only the initial signal to noise is different.

## Conclusion

Optical sectioning with SMFB has been reported in the literature. However, current methods dictate the use of complex modulated scanning, fluorescent staining, or sparse scatters. Here we showed how high-resolution SMFB can be used to image single cells and as complex a biological structure as the retina. We demonstrated how lensless sequential scanning of a sample using our SMFB makes it possible to image high spatial frequency features even through a ~360 µm diffusive layer with a scattering coefficient μ_s’_ of 1.3 [mm^−1^], while the imaging was acquired using non-coherent LED illumination.

The stitching algorithm helped diminish the noise level not just statistically, because there were more images, but also because it enhanced the static features in these images. Small displacements were made between each of the stitched images. The angle between the fiber cores and the sample changed slightly as well, since the fiber was located further from the sample (as the working distance allowed). Due to the angle, noises generated by light that were scattered from the diffusive medium randomly shifted their positions in the camera, whereas the valid signal from the sample did not shift. As a result, the contrast of the valid signal improved because stitching and interpolating enhanced the static signals, and diminished the non-static signals.

This method potentially enhances the capabilities of the proposed micro-endoscope device for use in minimally invasive procedures, because it allows features down to the size of the core to be resolved through a thin layer of biotissue. Few small steps in the order of tenths of microns are sufficient to introduce the data needed for the G-SR algorithm, and allow scattered noise reduction. This makes it feasible to perform a scan inside a living organism. Locating the fiber with a slightly wider hollow clad, the scan may be done by pulling and applying small vibrations on the fiber or just using natural vibrations. Stitching can then be done by image correlation or other techniques.

Our method does not suffer from the mechanical and optical limitations affecting in lens-based imagers. This allows for full flexibility in choosing the size and the shape of the region of interest to be scanned. The flexible fiber permits long-term scanning, which may be attractive for procedures and operations that take several hours thus competing with intraoperative OCT [31] and overcoming one of the main limitations on other imaging alternatives.

We believe that when operating within the visible regime, our technique can be combined with other methods such as fluorescence or phase-based methods (OCT). This may provide a new sensing capability for future imaging modalities in both fundamental as well as applied scientific research in cases where significant light scattering occurs.

## Abbreviations

FOV: field of view
GRIN: graded index
G-SR: geometrical super resolution
LED: light emitting diode
MMF: multimode fiber
NA: numerical aperture
OCT: optical coherence tomography
PDMS: polydimethylsiloxane
SMFB: single-mode fiber bundle
